# Clinicopathological Features and Metastatic Pattern of Triple-positive Breast Cancer Among Female Patients at a Tertiary Care Hospital

**DOI:** 10.7759/cureus.6458

**Published:** 2019-12-24

**Authors:** Walaa Alzahrani, Fatma Althoubaity, Dur Alsobhi, Yasmina Mohamed, Amani AlMutairi, Dalia Sindi, Rasha Alharbi, Nisar Zaidi

**Affiliations:** 1 General Surgery, King Abdulaziz University Hospital, Jeddah, SAU; 2 Surgery, King Abdulaziz University Hospital, Jeddah, SAU

**Keywords:** breast cancer subtype, triple positive breast cancer, clinical presentations, metastasis

## Abstract

Objectives

The heterogenicity of breast cancer (BC) is determined by the status of human epidermal growth factor receptor 2 (HER2/neu), estrogen receptor (ER), and progesterone receptor (PR). Triple-positive BC (TPBC) expresses the amplification/overexpression of the HER2 pathway and is positive for ER and PR. This subtype has a distinct clinical behavior. However, very few studies are focused on TPBC. This study investigated the clinicopathological features and metastatic pattern of TPBC.

Methods

A seven-year retrospective study was conducted at King Abdulaziz University Hospital in Jeddah, Kingdom of Saudi Arabia. All females with TPBC diagnosed between January 1, 2010, and June 30, 2017, were enrolled. Mean and standard deviation were calculated.

Results

From 1205 BC patients, the TPBC incidence was 10% (n = 124). The mean age at diagnosis was 51 years. On physical examination, a high tendency to show pathological skin changes was observed. Invasive ductal carcinoma was the most common histological type, presenting with a poorly differentiated histological grade (grade 3). Over a median two-year follow-up, the incidence of metastasis was 27.4% (n = 34). Bone was the most common site. The incidence of locoregional recurrence was 9.7%. Overall survival was 89.5%.

Conclusion

TPBC has an early tendency for metastasis and commonly affects the breast skin. BC should be approached based on the immunohistochemical diagnosis. We encourage more comprehensive studies to target TPBC for more insights into the heterogeneity of BC.

## Introduction

Breast cancer (BC) is considered globally to be the most common among females. A total of 1,700,000 new cases of BC were discovered in 2012, with a global standardized mortality rate of 12.9 per 100,000 [1]. BC in Saudi Arabia differs from that in other countries; it affects younger age groups and is being discovered at more advanced stages [2]. In 2010, there were 1473 cases of BC in Saudi Arabia with an age-standardized incidence rate of 24.9 per 100,000, which accounts for approximately 27.4% of all newly diagnosed female cancers in that year [3].

BC is not a one-entity disease; it is a heterogeneous malignancy with diverse molecular subtypes that are classified by the status of specific receptors detected by immunohistochemistry, namely, estrogen receptor (ER), progesterone receptor (PR), and human epidermal growth factor receptor-2 (HER2). Clinicians rely on the status of these receptors in their diagnosis, in making therapeutic decisions, and to determine survival rates [4]. In all, 75% of BC cases are positive for hormone receptors (HRs), either ER, PR, or both, and 20% show an overexpression/amplification of the HER2 pathway [5].

A new BC subtype has been considered from the HER2-enriched group, known as triple-positive BC (TPBC), which expresses the amplification/overexpression of the HER2 pathway and is positive for both ER and PR [4]. Clinical evidence has proven a distinct behavior [6]. Regarding the histopathological features of TPBC, patients with these tumors are shown to have higher tumor grade, have a larger tumor size, exhibit worse prognosis than other subtypes, and seem to have aggressive behavior [6]. Regarding the age of diagnosis for tumors that are hormonally positive, some data suggest that with increasing age, the incidence of hormonally positive tumors increases while the incidence decreases for triple-negative tumors [7]. TPBC is found to be resistant to chemotherapy and hormonal therapy due to numerous crosstalks between the two capital pathways, the ER and HER2 pathways. These crosstalks are responsible for the development of such resistance because blocking one pathway will upregulate the other one [8].

There are few studies in our region that have investigated the clinical behavior of this cancer. In this study, we investigated the clinicopathological features and metastatic pattern of TPBC among female patients diagnosed at King Abdulaziz University Hospital (KAUH), Jeddah, Kingdom of Saudi Arabia (KSA).

## Materials and methods

Study population

After the approval of the institutional review board at KAUH, a retrospective study was conducted at KAUH in June 2018. All files of any female subject, of any age, diagnosed at KAUH with primary BC, staged I-III, were electronically extracted from the KAUH electronic phoenix system for medical records using International Classification of Diseases, Tenth Revision (ICD-10) code C50.011- D05.82, diagnosed between January 1, 2010, and June 30, 2017. A total of 1205 primary BC female cases were identified. We then included only those who are proven to be TPBC, and we excluded the other subtypes. The identification of TPBC cases was done by referring to the histopathology reports, the immunohistochemistry stain for the status of ER/PR and HER2/neu of each file was checked as detailed below.

ER/PR staining

If the percentage of staining for tumor cell nuclei was >20%, the receptor status is considered positive. The receptor status is considered negative if staining was <5%. Between 5% and 19% is a borderline and, for the sake of this study, both positive results and borderline results were included as positive.

HER2/neu

The result of HER2/neu is reported using a scoring system. Score +3 corresponds to positive HER2, score +2 is borderline, and score +1 to 0 is negative. Only score +3 was regarded as positive, and for score +2, a subsequent verification by fluorescent in-situ hybridization (FISH) was checked to include only positive FISH.

Definition of intrinsic subtypes

According to the recommendations of the St. Gallen Conference in 2011 for defining BC subtypes with an immunohistochemical status and proliferation activity [9], in our study, we used the tumor grade as an indicator of proliferation activity because determining Ki67 status was not a standard in our hospital. By using tumor grade to capture proliferation activity as described earlier, the subtypes were defined as the following: luminal A (ER+, HER2-, G1/2), luminal B (ER+, HER2-, G3), triple-positive (ER+, PR+, HER2+), HER2-enriched (ER-, HER2+), and triple-negative (ER-, PR-, HER2-) [10].

Data collection and statical analysis

By using medical records, we obtained the following: site of the disease, clinical presentation, demographic information, age at diagnosis, histopathological features, history of metastasis or recurrence, and the performed interventions. Data entry was achieved using Microsoft Excel 2014 and statistical analysis was performed by Statistical Package for Social Science Version 21 (Armonk, NY: IBM Corp.) using median, mean, and standard deviation (SD) for quantitative data and frequency for qualitative data.

## Results

**Figure 1:**
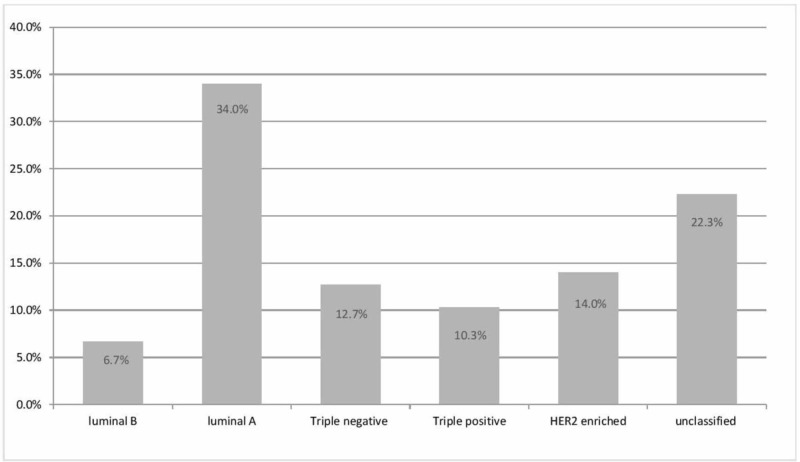
Distribution of molecular subtypes of breast cancer at KAUH according to the St. Gallen 2011 classification

The study aimed to explore the clinical features of TPBC. Over a seven-year period from January 1, 2010, to June 30, 2017, 1205 primary BC female cases were identified in KAUH. After the stratification of these 1205 cases via the status of the receptors using immunohistochemistry results from the pathology reports, only 124 were regarded as TPBC. These represented 10.3% of the total cases. According to the St. Gallen classification of BC, the subtype frequency was as follows: the most common was luminal A with 409 cases (34%), followed by HER2-enriched with 169 cases (14%), triple-negative BC with 153 cases (12.7%), TPBC with 124 cases (10.3%), and the least common was luminal B, with 81 cases (6.7%). There were 269 cases (22.3%) proven to be BC but the immunohistochemical classification was incomplete/not carried out. Figure 1 illustrates the distribution of the molecular subtypes among the KAUH female patients diagnosed with BC.

After obtaining the frequency of TPBC, the median time of follow-up was 25 months (range of 0.6-145 months). The median age at diagnosis was 49 years and the mean age at diagnosis was 51 years (SD: 12.7, range of 25-93 years). As for their baseline health status, we found out that most of them were overweight/obese (76.9%) and 23.1% had normal body mass index (BMI). Along with BC, exactly half of the cases had co-existing chronic comorbidity while the other half were medically free at the time of presentation (n = 63, 50%). Co-existing malignancies other than BC were identified only in 4.8% of the cases (n=6). The most common complaint of TPBC patients was the presence of a breast mass discovered by a self-breast examination or accidentally upon a physical examination (n = 84, 67.7%) rather than pain, which occurred only in 10 cases (8.1%), and nipple discharge was not reported (n = 0). The most common clinical sign at presentation was pathological skin changes, whether in the form of skin tethering, thickness, erythema, pigmentation, or ulcerations. Table 1 illustrates the demographic data, baseline health status, and clinical presentations.

**Table 1:** Clinical features of triple positive breast cancer patients BMI: body mass index

Variable	No. of subjects (%) (n = 124)
Mean age at diagnosis (range)	51 ± 12.7 (25–93)
Age at diagnosis (years)	
≤ 50	64 (51.6%)
< 50	60 (48.4%)
Mean BMI kg/m^2^ (range)	29.59 ± 6.8 (16.23–54.05)
Underweight (> 18.5)	0
Normal weight (18.5–24.9)	24 (23.1%)
Overweight (25–29.9)	37 (35.6%)
Obese (30–39.9)	36 (34.6%)
Morbidly obese (< 40)	7 (6.7%)
Co-existing chronic co-morbidity	
Yes	62 (50%)
No	62 (50%)
Type of chronic co-morbidity	
Hypothyroidism	10 (8.1%)
Diabetes	24 (19.4%)
Hypertension	32 (25.8%)
Cardiac diseases	24 (19.4%)
Chronic liver disease	3 (2.4%)
Chronic kidney disease	1 (0.8%)
Chronic neurological diseases	6 (4.8%)
Chronic lung diseases	6 (4.8%)
Co-existing cancer	
Yes	6 (4.8%)
No	118 (95.2%)
Type of co-existing cancer	
Colon cancer	2 (1.6%)
Leukemia	2 (1.6%)
Thyroid cancer	1 (0.8%)
Endometrial cancer	1 (0.8%)
Symptoms at presentation	
Mass	84 (67.7%)
Pain	10 (8.1%)
Nipple discharge, Signs at presentation	0 (0%)
Morphological changes	29 (23.4%)
Skin changes	51 (41.1%)
Nipple changes	20 (16.1%)

**Figure 2:**
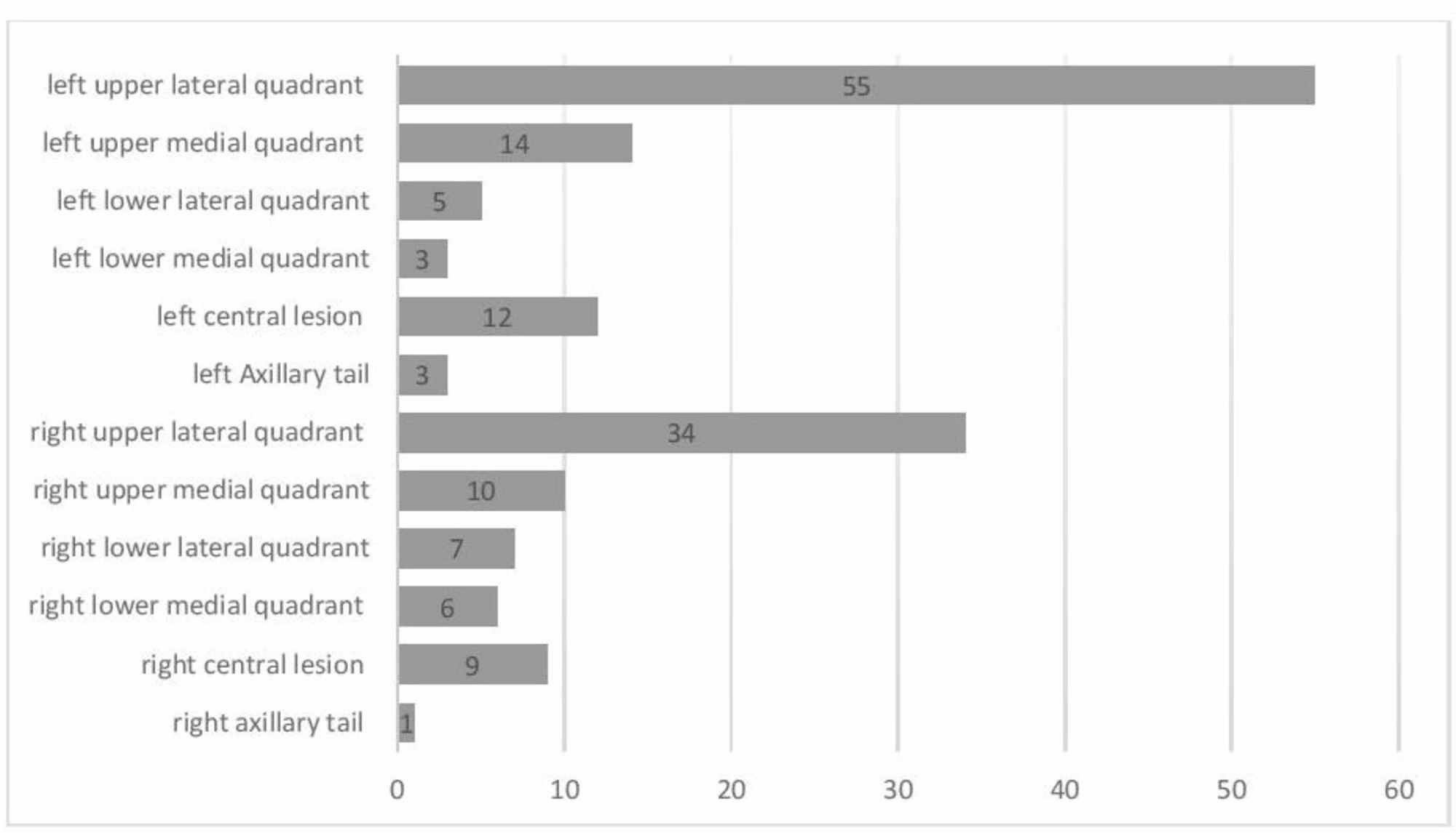
Sites of triple-positive breast cancer at diagnosis

The left upper outer quadrant was the most common site of TPBC at diagnosis (n = 55, 34.6%), followed by the right upper outer quadrant (n = 34, 21.4%). Tumors were less likely to be found at the right axillary tail (n =1, 0.6%). Figure 2 illustrates the tumor locations at the time of diagnosis.

Histopathological features

Table 2 demonstrates the baseline histopathological characteristics of the TPBC cases. The most common pathological type was invasive ductal carcinoma (n = 100, 80.6%) with a ductal carcinoma in situ component co-existing with invasive BC 69.4% of the time (n = 86). We also identified seven cases of TPBC exhibiting Paget disease of the nipple as well (5.6%). The subtype of ductal carcinoma was not otherwise specified in 45.2% of the cases. However, the solid pattern was the most frequently observed pattern and the papillary pattern was the least (n = 50, 40.3% and n = 3, 2.4%).

**Table 2:** Histopathological features of triple-positive breast cancer

Variable	No. of subjects (%) (n = 124)
Tumor size (cm)	
≤ 2	43 (34.7%)
2.1–5	53 (42.7%)
< 5	17 (13.7%)
Missing	11 (8.9%)
Pathological type	
Ductal carcinoma in situ	86 (69.4%)
Invasive ductal carcinoma	100 (80.6%)
Lobular carcinoma in situ	9 (7.3%)
Invasive lobular carcinoma	9 (7.3%)
Medullary carcinoma	2 (1.6%)
Mucinous carcinoma	2 (1.6%)
Subtype of ductal carcinoma	
Not otherwise specified	56 (45.2%)
Comedo	47 (37.9%)
Cribriform	37 (29.8%)
Papillary	3 (2.4%)
Micropapillary	11 (8.9%)
Solid	50 (40.3%)
Not mentioned	22 (17.7%)
Histological grade	
Well-differentiated	9 (7.3%)
Moderate differentiation	40 (32.3%)
Poor differentiation	73 (58.9%)
Missing	2 (1.6%)
Nuclear pleomorphism: Grade 1, Grade 2, Grade 3, Unknown	8 (6.5%), 71 (57.3%), 43 (34.7%), 2 (1.6%)
Mitotic figures	
Score 1	70 (58.5%)
Score 2	31 (26.7%)
Score 3	15 (12.1%)
Unknown	8 (6.5%)
Lymphatic invasion	
Positive	40 (32.3%)
Negative	66 (53.2%)
Not examined	18 (14.5%)
Type of positive lymphatic invasion	
Lymphovascular	36 (90%)
Lymphodermal	1 (2.5%)
Both	3 (7.5%)
Neuronal invasion	
Yes, No	11 (8.9%), 113 (91.1%)
Surgical Margin	
Positive	19 (15.3%)
Negative	105 (84.7%)
Median number of lymph node involvement (range)	1 (0–23)
Skin involvement	
No involvement	105 (84.7%)
Dermis only	3 (2.4%)
Epidermis only	2 (1.6%)
Subcutaneous fat	3 (2.4%)
Skin	4 (3.2%)
Paget disease	7 (5.6%)

The tumors had a median size of 2.7 cm. At presentation, most of the tumors had poorly differentiated histological grade (n = 73, 58.9%), moderate nuclear pleomorphism (n=71, 57.3%), and low mitotic figures (n=70, 58.5%).

Lymphatic invasion was negative in most cases (n = 66, 53.2%). It was identified in 40 cases (32.3%) and was lymphovascular rather than lymphodermal (n = 36, 90% and n = 1, 2.5%). The mean number of lymph nodes that showed tumor deposition was 3 ± 5 (range 0-23). Histopathological evidence of skin invasion shows that skin was spared from the tumor cell in most cases (n = 105, 84.7%).

Locoregional reoccurrence and distant metastasis

Over a median follow-up period of 25 months, the incidence of distant metastasis was 27.4% (n = 34); 38.2% of the metastatic cases died (n = 13). Twelve (35.3%) presented as stage 4 at the time of diagnosis considered as primary metastatic cases and 22 (64.7%) underwent secondary metastasis later on.

For secondary metastatic cases, the mean time from diagnosis to metastasis was 32.3 ± 31 months and the median time was 26.2 months (range 1.2-102 months).

The most common site of TPBC metastasis was the bone (n = 23, 67.6%), followed by the lung (n = 18, 52.9%). The incidence of locoregional recurrence over a median follow-up period of 25 months was 9.7% (n = 12). The average duration from diagnosis to recurrence was 38.6 ± 30.8 months (range 7-108 months). The overall 25 months' survival was 89.5%. The metastatic pattern of TPBC is illustrated in Table 3.

**Table 3:** Metastatic patterns

Variables	No. of subjects (%) (n = 34)
Metastatic cases	34 (27.4% )
Mean age at metastasis in years	51 ± 14
Mortality among metastatic cases	13 (38.2%)
Primary metastasis	12 (35.3%)
Secondary metastasis	22 (64.7%)
Median duration from diagnosis until metastasis for secondary cases (months)	26.2
Site	
Bone	23 (67.6%)
Lung	18 (52.9%)
Liver	13 (38.2)
Pancreas	1 (2.9%)
Spleen	2 (5.9%)
Intestinal tract	1 (2.9%)
Ovaries	3 (8.8%)
Brain	9 (26.5%)
Skin	1 (2.9%)
Contralateral breast	5 (14.7)

Therapeutic approaches

A total of 88.7% of the cases had surgery. The most common surgical intervention performed for TPBC was modified radical mastectomy rather than conservative breast therapy. Of those who underwent surgery, 14.5% had postoperative complications as follows: seroma in 9.7%, cellulitis in 2.4%, hematoma in 1.6%, prolonged breast pain in 1.6%, and lymphedema in 1.6%. Of these, 16.9% had to go for another surgical intervention in the same breast. Table 4 summarizes the attempted interventions.

**Table 4:** Types of interventions

Variable	No. of subjects (%) (n = 124)
Primary surgical intervention	110(88.7%)
Modified radical mastectomy/Mastectomy	69 (63%)
Lumpectomy with axillary dissection	23 (21%)
Lumpectomy with sentinel lymph node biopsy	18 (16%)
Secondary surgical intervention	21 (16.9%)
Reconstructive/debridement	8 (40%)
Re-lumpectomy	3 (15%)
Modified radical mastectomy	10 (45%)
Radiotherapy	73 (58.9%)
Adjuvant chemotherapy	88 (7.1%)
Neoadjuvant chemotherapy	47 (37.9%)
Target chemotherapy (Trastuzumab)	64 (51.6%)
Hormonal therapy	95 (76.6%)

## Discussion

BC has a heterogeneous biological behavior and is considered as a complex disease with regards to treatment and prognosis [11]. The molecular classification of BC based on gene expression divides BC into five types: (1) luminal A (HR+, HER2-, and lower proliferation index), comprising 30%-40% of all BC, (2) luminal B (low ER+, PR-, HER2+, and high proliferation index), comprising 30%-40% of all BC, (3) HER2+ and HR-, comprising 15%-25% of all BC, (4) basal-like triple-negative, comprising 10%-20% of all BC, and (5) Claudin-low, which is usually triple-negative and comprises 10%-15% of all BC. A new subtype has been considered, which is positive for ER, PR, and HER2 showed distinct behavior in terms of the progression of the disease and the response to chemotherapy and hormonal therapy. This subtype was called TPBC. TPBC has a higher grade, larger size, and worse prognosis when compared to triple-negative BC (Ades et al., 2014) [4,6,12-13]. It has been reported that HER2-positive cases have lower overall survival and a worse prognosis as compared to cases in which HER2 is negative without target therapy, and when the patient has HER2-positive/ER-positive, the optimal therapy sequence remains unclear [4,14-15] The effectiveness is still questionable for the targeted therapy when dealing with TPBC, trastuzumab against HER2, and anti-estrogen against ER. It has been proposed that crosslinked between the two capital pathways will result in resistant of targeted therapy compared with only one positive pathway and this still needs to be extensively studied [8]. In our study, the mean age at diagnosis was 51 years, which is similar to the study by Negi et al., where it was 49.6 years [6]. In our study, 48.6% of patients were above 50 years of age, which contrasted with the findings of Negi et al., where 65% of the patients were above 50 years of age. This can be attributed to the fact that our patients sought medical advice earlier due to the availability of tertiary health care to them, which led to prompt diagnosis and treatment. In our study, 10.2% of 1205 patients had TPBC while Juan et al. reported the incidence of TPBC to be 18.2% out of 13,264 patients [16]. Other studies by Ali et al., Voduc et al., and Cary et al. reviewed the incidence of TPBC to be between 9.5% and 20% [17-19].

When we observed the symptoms at presentation, we found that most patients presented with typical symptoms of BC regardless of the molecular subtype. The most common symptom of TPBC was a breast mass discovered accidentally (n = 84, 67.7%) rather than pain, which happened in a minority of cases (n=10, 8.1%), and nipple discharge was never reported (n = 0). The frequent site in which TPBC was found in was the left upper outer quadrant, which concludes that TPBC does not have atypical symptoms, such as a higher incident of pain/tenderness or a higher incident of nipple discharge as an initial presentation, nor does it have a tendency to present more frequently in a different quadrant than what typical BC presents with. The most common site of breast cancer, regardless of the molecular subtype, is the left upper outer quadrant, and the most common symptoms at presentation is a breast lump with breast pain and nipple discharge rarely point to BC as an initial presentation [20-21]. What we found interesting was that our data showed a high tendency of TPBC to present with pathological skin changes discovered during the time of physical examination in terms of breast ulceration, erythema, pigmentation, or thickness (n=51, 41.1%). Moreover, we found an unusually high incidence of Paget disease, which occurred with 5.6% of TPBC; meanwhile, Paget disease represents only 1%-3% of all BC regardless of the molecular subtype [22].

Regarding their baseline health status, obesity may play a role in BC, which is shown in a study by Adam et al., and we found that 76.9% of TPBC were overweight/obese [23]. In our study, almost half of the patients had co-morbidities and the commonest was hypertension (25.8%), which is similar to the study by Sharma et al., where they reported the incidence of hypertension among BC patients to be 21.8%, and they stated that hypertension is the most prevalent comorbidity associated with BC [24]. However, we could not associate co-morbidities with TPBC, as the other half of TPBC besides having BC were medically free. Metastasis occurred in 27.4% of the cases, a study by Kennecke H et al. stated that TPBC had the highest tendency of metastasis among the other molecular subtypes [25]. In our study, bone metastasis occurred in 67% of the cases, which is similar to a study that showed that HR+ tumors mostly metastasized to the bone while HR- tumors metastasized to multiple visceral sites [26]. However, TPBC showed an early tendency for metastasis as 27.4% of our cases reported metastasis over a median follow-up period of 25 months. This was opposite to what we found in the literature, where it was reported by Ahmed et al. that early metastasis occurred in HR- cancer while TPBC required 15 years to metastasize [27]. This emphasized the fact that we should follow up on our patients for metastasis more closely.

Regarding the primary surgical procedures that were carried out, the majority of TPBC cases underwent modified radical mastectomy (63%) rather than breast conservative therapy (37%), which indicated that the disease had more advanced clinical features at presentation, which did not fulfill the criteria for breast conservative therapy.

Our study has an important clinical implication in terms of shedding light on the importance of following patients who are diagnosed with the TPBC subtype more closely for metastasis due to the tendency for early metastasis, as illustrated by the short duration between diagnosis and metastasis among the metastatic cases.

The limitation of this study was that among a total of 1205 primary BC cases, we had 15 files in which the HER2 results were scored as a score of 2+, but with no subsequent fluorescent in-situ hybridization (FISH) analysis, they were regarded as borderline cases and were included as HER2-positive cases. Moreover, our hospital system’s pathology reports had a total of 269 cases (22%) proven to be BC but with missing or incomplete immunohistochemical staining, which prevented a complete classification of all breast cancer cases.

## Conclusions

In conclusion, BC should always be approached according to the status of its receptors and emphasis should be given to the early diagnosis and treatment of TPBC to prevent the morbidity and mortality associated with Its tendency for early metastasis.
